# Pan-tumor validation of FoundationOne^®^CDx as a companion diagnostic for *RET* fusions as a predictor of response to selpercatinib

**DOI:** 10.3389/fonc.2026.1820286

**Published:** 2026-05-13

**Authors:** Allison van den Hout, Richard S. P. Huang, Caleb Ho, David L. Smith, Douglas A. Mata, Baljinder Kaur, Sameera R. Wijayawardana, Jennifer Wright, Anna M. Szpurka, Douglas I. Lin

**Affiliations:** 1 Foundation Medicine, Inc., Boston, MA, United States; 2 Eli Lilly and Company, Indianapolis, IN, United States

**Keywords:** companion diagnostic (CDx), comprehensive genomic profiling (CGP), FoundationOne^®^CDx, RET fusion, validation

## Abstract

**Background:**

*RET* fusions represent a subgroup of alterations in cancer biology that exhibit sensitivity to selective RET kinase inhibitors. We analytically validated a next-generation sequencing-based companion diagnostic (CDx) to detect *RET* fusions in solid tumors via a comprehensive genomic profiling assay, FoundationOne^®^CDx (F1CDx^®^).

**Methods:**

The F1CDx assay produces chimeric read pairs to determine fusion status. Additionally, a study of F1CDx was conducted as a retrospective clinical bridging study based on LIBRETTO-001 clinical trial samples and data.

**Results:**

Across solid tumor types, F1CDx demonstrated a high concordance for *RET* fusion detection with both an externally validated assay and LIBRETTO-001 clinical trial assays with positive percent agreements of 100.0% (146/146) and 90.80% (79/87), negative percent agreements of 97.3% (254/261) and 100.0% (138/138), and overall percent agreements of 98.2% (400/407) and 96.44% (213/225), respectively. Based on LIBRETTO-001 clinical data, the objective response rate to selpercatinib for patients with tumors harboring *RET* fusions as determined by F1CDx was estimated to be 69.62%. Demonstrated analytical and clinical performance of F1CDx led to the pan-tumor approvals from the U.S. Food and Drug Administration (FDA) in 2023 and the Japan Ministry of Health, Labour and Welfare (MHLW) in 2024 of F1CDx to identify *RET* fusions in solid tumor patients for treatment with selpercatinib.

**Conclusions:**

F1CDx is an accurate, reliable, FDA- and MHLW-approved method for the pan-tumor identification of *RET* fusions for treatment with selpercatinib.

## Introduction

The rearranged during transfection (*RET*) gene, located near the centromere on the long arm of chromosome 10 (10q11.21), encodes a receptor tyrosine kinase (RTK) with roles in organogenesis and in the maintenance of several adult tissue types, including neural, neuroendocrine, hematopoietic, and male germ cell tissues (1). In addition, *RET* is a proto-oncogene, and activating somatic *RET* alterations, including mutations and fusions, are oncogenic driver alterations in approximately 2-10% of solid tumors, depending on the tumor type (2). *RET* fusion has emerged as a biomarker for advanced cancer patients with solid tumors who may benefit from selpercatinib and who have progressed on or following prior systemic treatment or who have no satisfactory alternative treatment options.

Multiple lines of evidence suggest that *RET* fusions are activating genomic events leading to oncogene addiction regardless of the tumor type in which they arise. *RET* fusions promote cell proliferation and survival in human cancer models and exhibit oncogene addiction—a hallmark of driver alterations. In patient-derived models harboring *RET* fusions, selective RET inhibition induces tumor cell death, highlighting *RET* fusions as predictive biomarkers for identifying patients who may benefit from targeted RET-directed therapies (2). *RET* gene fusions occur most commonly in lung cancer (~1% - 2% non-small cell lung cancer (NSCLC)),and papillary thyroid cancer (~5-10% PTCs), and in extremely rare subsets of other cancers, including breast, colon, esophageal, ovarian, prostate, stomach, pancreatic, salivary gland cancers, and sarcomas (most occurring at rates of <1%) (3–6. 7).

*RET* fusions may be detected via different techniques in formalin-fixed, paraffin-embedded (FFPE), solid tumor tissues, such as via fluorescent *in-situ* hybridization (FISH) or DNA- or RNA-based next-generation sequencing (NGS). In contrast to NGS-based comprehensive genomic profiling (CGP), FISH does not provide additional context of the gene fusion such as partner gene identification and does not include other NCCN-recommended NGS-based biomarkers in relevant cancer types.

The FDA and MHLW have both recently approved an NGS-based assay, FoundationOne^®^CDx (F1CDx^®^; Foundation Medicine, Inc., Boston, MA), as a companion diagnostic (CDx) to identify patients with *RET* fusion-positive solid tumors for treatment with selpercatinib. F1CDx is a CGP assay that assesses oncogenic alterations involving 324 genes as well as complex biomarkers, such as microsatellite instability (MSI), tumor mutational burden (TMB), and homologous recombination deficiency signature (HRDsig), the latter offered as a laboratory professional service. Here, the robust pan-tumor analytical and clinical validity of F1CDx for the identification of *RET* fusion-positive solid tumor patients is demonstrated, which led to the FDA and MHLW approvals of F1CDx as a CDx for selpercatinib.

## Methods

### F1CDx assay

F1CDx is an FDA- and MHLW-approved, NGS-based CGP, *in vitro* diagnostic device based on hybridization-based capture technology and NGS for the detection of substitutions, insertion and deletion alterations, copy number alterations, and select rearrangements in 324 genes as well as microsatellite instability (MSI) and tumor mutational burden (TMB). F1CDx methods have been described previously (8). Sheared genomic DNA (50 to 1,000 ng) isolated from FFPE tumor tissue specimens are used for library construction (LC). Libraries of 500 to 2,000 ng of DNA undergo solution hybridization using a pool of individually synthesized 5’-biotinylated DNA 120-bp oligonucleotides to select target sequences. After amplification and purification, captured libraries are sequenced using off-board clustering with patterned flow cell technology to generate monoclonal clusters from a single DNA template followed by sequencing by synthesis on the Illumina platform (Illumina, Inc., San Diego, CA). Sequencing data were analyzed using Foundation Medicine’s proprietary bioinformatics pipeline to identify base substitutions, short insertions/deletions (indels), copy number alterations, and genomic rearrangements. Variant calling is performed as previously described (9).

### Rearrangement detection

Genomic rearrangements are identified through analysis of chimeric read pairs. Chimeric read pairs are defined as paired-end reads in which two reads mapped either to different chromosomes or to positions greater than 10 megabase (Mb) apart on the same chromosome. Chimeric read pairs were clustered by genomic coordinates, and clusters containing at least five chimeric pairs (or at least three for rearrangements involving known fusions) were designated as candidate rearrangements.

Candidate rearrangements were further evaluated based on mapping quality and alignment distribution. Clusters were required to have an average read mapping quality **≥**30, and clusters with non-uniform alignment distributions suggestive of mapping artifacts were excluded. High-confidence rearrangements were annotated for predicted function consequence, including the potential creation of fusion genes (10).

### Analytical validation

To demonstrate the F1CDx assay’s ability to detect *RET* fusions, several analytical validation studies were conducted: limit of blank (LoB), limit of detection (LoD), site-to-site precision, and orthogonal concordance. As selpercatinib is FDA- and MHLW-approved as a kinase inhibitor indicated for treatment of adult patients with locally advanced or metastatic solid tumors, a variety of pan-tumor samples were selected for the analytical validation studies to represent the intended use population. Fusions in *RET* were considered CDx biomarker positive in the analytical validation studies if the following conditions were met: in-strand rearrangement events that lead to a *RET* fusion with another protein coding gene in which the *RET* kinase domain is not disrupted and *RET* is on the 3′ end of the detected fusion.

### Limit of blank

LoB studies were executed to assess the LoB for *RET* fusions in 33 total samples, which included six NSCLC, six thyroid cancer (TC), and 21 other solid tumor samples.

### Limit of detection

**Figure 1:**
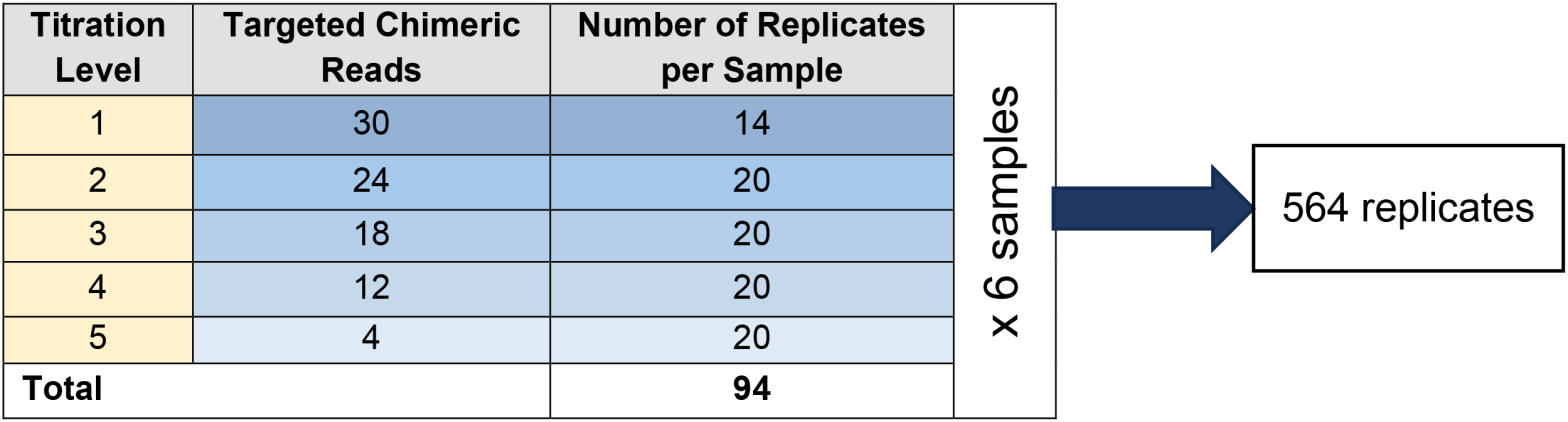
Sample replicates in the LoD study design.

An LoD study was executed to establish the detection limit of F1CDx for *RET* fusions using six different samples. A representative approach to select samples with a variety of *RET* fusion partners and different cancer types was considered for this solid tumor indication. Each sample was assessed at five titration levels (30, 24, 18, 12, and 4 targeted chimeric reads). For each sample, a total of 94 replicates were processed; 20 replicates were processed for each dilution level, except at the 30 chimeric reads level for which 14 replicates were processed to retain all 94 replicates within one plate (**Figure 1**). A total of 564 replicates were processed for the study. Biomarker-negative samples (i.e., diluent DNA) were selected to dilute the biomarker-positive samples to establish the five different titration levels. Biomarker-negative samples from the same tumor type were used to dilute the chimeric read counts.

Samples with approximately 50 nanograms of DNA were processed, which is the minimum input requirement for F1CDx and represents the most challenging conditions for fusion detection.

### Precision (reproducibility and repeatability)

**Figure 2:**
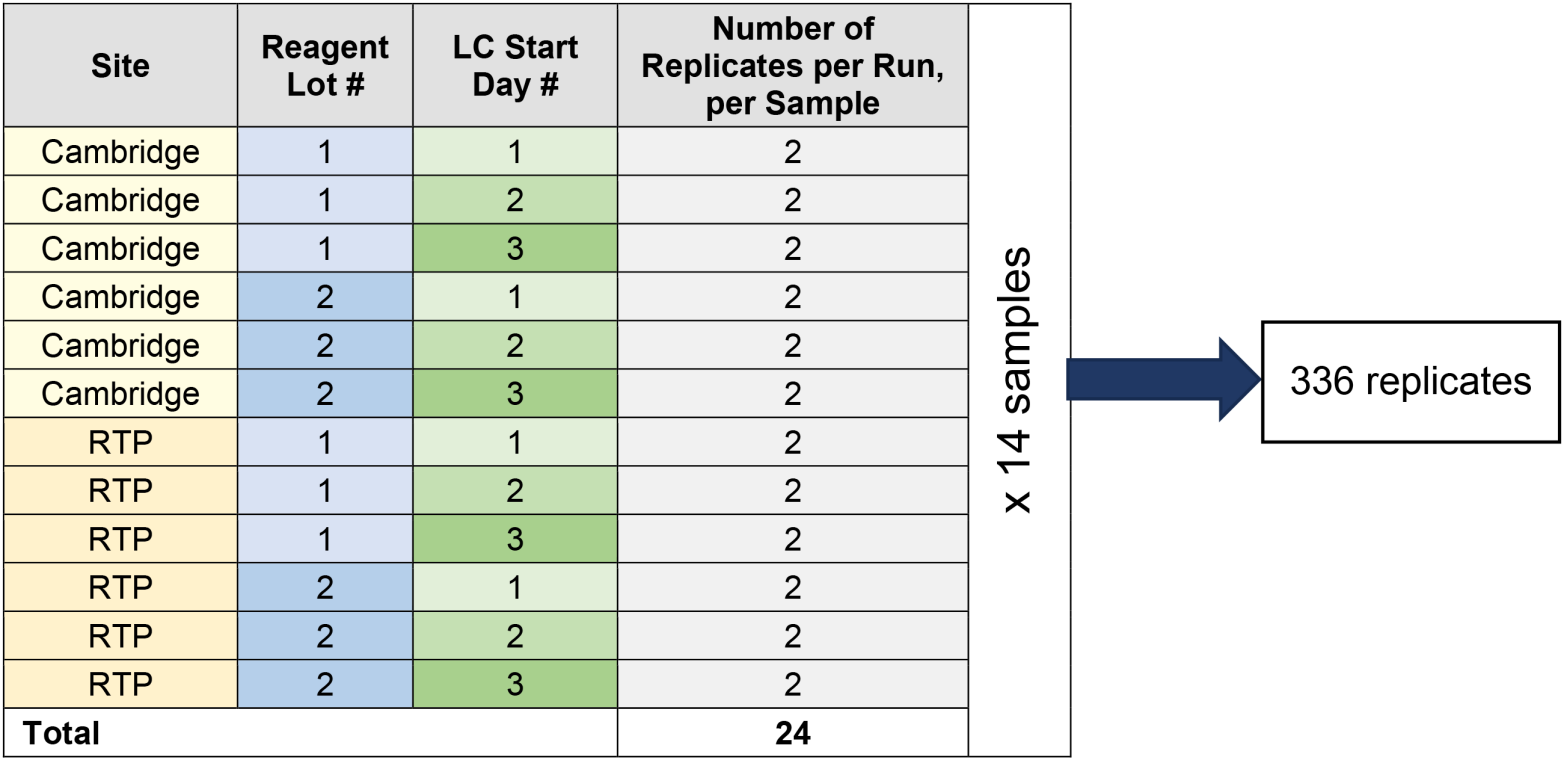
Sample replicates in the precision study design.

A site-to-site precision study was performed to evaluate the reproducibility and repeatability of biomarker-positive samples using 14 source samples from a range of tumor types. Each source sample was diluted with *RET* fusion-negative diluent samples from the same tumor type to achieve the desired LoD range of 1-3x the LoD (fold LoD) established for *RET* fusions. Each source sample was tested in two (duplicate) replicates at two sites (Cambridge, MA and Research Triangle Park (RTP) in Morrisville, NC), on three LC start days, with two reagent lots, for a total of 24 replicates (**Figure 2**). Altogether, 336 replicates of source samples containing *RET* fusions were processed in this study.

### Concordance with orthogonal assays

**Figure 3:**
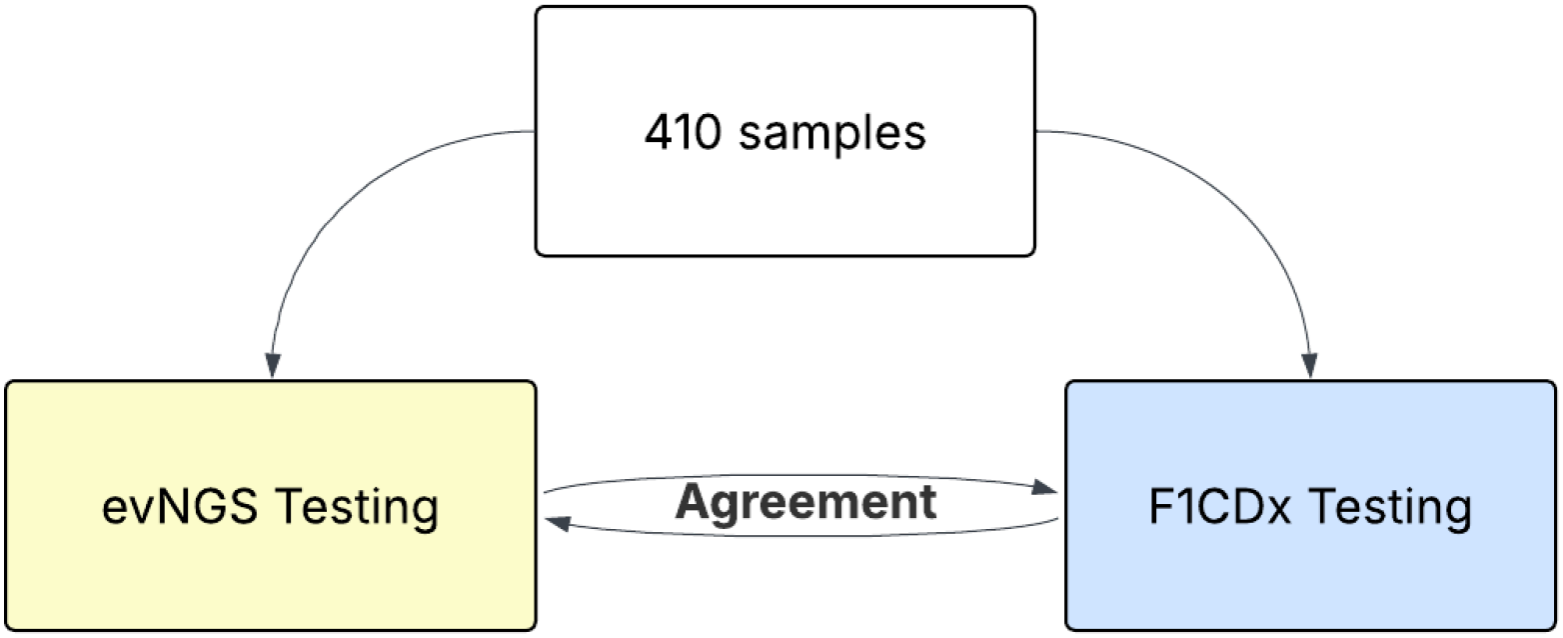
Samples in the orthogonal concordance study design.

A concordance study was conducted to evaluate the concordance of *RET* fusion detection in patients with solid tumors by F1CDx and an orthogonal assay. The study was performed with 31 samples from patients with solid tumors enrolled in LIBRETTO-001, that supported the selpercatinib approval, plus 125 *RET* fusion-positive and 254 *RET* fusion-negative samples from FMI’s clinical archives or previous FMI’s validation studies, for a total of 410 samples. The orthogonal assay used for concordance testing was an externally validated NGS (evNGS) assay that is performed in a College of American Pathologists (CAP)-accredited, Clinical Laboratory Improvement Amendments (CLIA)-certified laboratory. To assess the concordance of variant calls, positive percent agreement (PPA), negative percent agreement (NPA), positive predictive value (PPV), and negative predictive value (NPV), were estimated (**Figure 3**).

### Clinical bridging study

The clinical utility of F1CDx for the detection of *RET* fusions in patients with solid tumors who may benefit from treatment with selpercatinib was demonstrated by a clinical bridging study, retrospectively testing FFPE tumor tissue specimens from *RET* fusion-positive solid tumor patients enrolled in the LOXO-RET-17001 (LIBRETTO-001, NCT03157128) clinical study and *RET* fusion-negative solid tumor patient samples from the FMI archives with F1CDx. The LIBRETTO-001 clinical study is an open-label, multi-center Phase 1/2 study in patients with advanced solid tumors, including *RET* fusion-positive solid tumors (e.g., NSCLC, thyroid, pancreas, colorectal), *RET*-mutant MTC, and other tumors with *RET* activation (e.g., mutations in other tumor types or other evidence of *RET* activation) (11, 12). The clinical study included two parts: Phase 1 (dose escalation and dose expansion) and Phase 2 (dose expansion). The primary objective of the Phase 1 portion of the study was to determine the maximum tolerated dose (MTD)/recommended Phase 2 dose (RP2D) of selpercatinib. RP2D was determined to be 160 mg of selpercatinib orally twice daily (BID). Primary efficacy, the primary objective of Phase 2, was measured by the objective response rate (ORR) using Response Evaluation Criteria in Solid Tumors (RECIST 1.1) as assessed by independent review committee (IRC).

The main objectives of the clinical bridging study were to evaluate the concordance between the local clinical trial assays (CTAs) used for trial enrollment and F1CDx in detecting *RET* fusions and to estimate the efficacy of selpercatinib among *RET* fusion-positive patients as determined by F1CDx, using samples and clinical data from the LIBRETTO-001 clinical trial. The efficacy assessment was performed with the pooled set (n=243) of *RET* fusion-positive NSCLC (n=169), TC (n=22), and tissue agnostic (TA) (n=52) patient populations, as identified by CTAs, used for the drug approval in the respective indications. The concordance assessment was performed with the pooled set of efficacy-evaluable patients, plus 140 procured samples that were determined *RET* fusion negative by a comparator NGS test (considered as CTA negative) and selected for the bridging study.

## Results

### Limit of blank and limit of detection

The LoB for *RET* fusions was demonstrated in the F1CDx assay by testing matched normal samples from patients with solid tumors. Thirty-three (33) solid tumor FFPE and peripheral blood mononuclear cells (PBMC) samples comprised the following diseases and specimen types: lung squamous cell carcinoma, lung non-small cell lung carcinoma, and lung adenocarcinoma (n=14 lung), thyroid follicular carcinoma, thyroid papillary carcinoma, and thyroid medullary carcinoma (n=6 thyroid), ovary serous carcinoma (n=5 ovary), breast invasive ductal carcinoma (n=2 breast), anus carcinoma (n=1 anus), bladder carcinoma (n=1 bladder), colon adenocarcinoma (n=1 colon), kidney clear cell carcinoma (n=1 kidney), skin sarcoma (n=1 skin sarcoma), and uterus endometrial adenocarcinoma (n=1 endometrial). Each sample was assessed in replicates of four to 15, resulting in a total of 345 replicates. Of the 345 replicates, 331 were successfully sequenced and passed the F1CDx post-sequencing quality control (QC) criteria and were included in the analysis. Among the 331 valid sample replicates, no *RET* fusions were detected, resulting in a false positive rate of 0%, defining the LoB for *RET* fusions as zero (**Table 1**).

**Table 1:** False positive rate of *RET* fusions in solid tumor samples.

Number of sample replicates with positive calls/ number of total sample replicates	False positive rate (%)
0/331	0	

The LoD for *RET* fusions was established for F1CDx in five of the six samples that demonstrated a monotonic relationship between the dilution levels and hit rates (positive call rates). Selection of specimens for assessment of *RET* fusions represented different *RET* fusion partner genes and various tumor types, including lung adenocarcinoma (n=2 lung), thyroid papillary carcinoma (n=1 thyroid), breast invasive ductal carcinoma (n=1 breast), and colon adenocarcinoma (n=1 colon). The LoD for each *RET* fusion was established within each of the five samples using the hit rate method and ranged from 4.90 to 10.85 chimeric reads (**Table 2**). The final LoD for *RET* fusions was 8.75 chimeric reads and was determined by using the median LoD for *RET* fusions from the five samples for which the LoD was established.

**Table 2:** Summary of LoD analysis for *RET* fusions.

Sample	Cancer type	*RET* fusion	LoD by hit rate method (chimeric reads)
1	Thyroid papillary carcinoma	*CCDC6::RET*	4.90	
2	Lung adenocarcinoma	*TRIM24::RET*	8.50	
3	Colon adenocarcinoma	*CCDC6::RET*	8.75	
4	Breast invasive ductal carcinoma	*KIF5B::RET*	10.80	
5	Lung adenocarcinoma	*ERC1::RET*	10.85	

### Precision (reproducibility and repeatability)

A site-to-site precision study was conducted to evaluate the reproducibility and repeatability of *RET* fusion detection and chimeric reads evaluation in 14 samples near the LoD and comprised the following disease and specimen types: thyroid carcinoma, thyroid papillary carcinoma (n=3 thyroid), ovary serous carcinoma (n=1 ovary), pancreas neuroendocrine carcinoma (n=1 pancreas), lung adenocarcinoma (n=3 lung), and colon adenocarcinoma (n=6 colon). For each sample, 24 replicates were processed, and at least 22 were successfully sequenced and passed the F1CDx post-sequencing QC criteria and included in the analysis. Reproducibility was evaluated by processing replicates from the same source sample under conditions where one variable was changed at a time (e.g., site, reagent lots, LC start days). To be considered a positive call, the *RET* fusion had to be detected in each replicate of the sample and meet the biomarker definition for *RET* fusions. Repeatability was evaluated by processing duplicate replicates from the same sample and plate within each site, reagent lot, and LC start day. The result was considered in agreement if the duplicate replicates processed under identical conditions had the same detection status for the *RET* fusion. For all 14 samples, the reproducibility of *RET* fusion detection near LoD was ≥95% and the repeatability was ≥90%. **Table 3** shows the reproducibility, repeatability, and cancer types for *RET* fusions in each of the 14 samples included in the precision study.

**Table 3:** Reproducibility and repeatability for *RET* fusion detection.

Sample	Cancer type	*RET* fusion	Fold LoD*	Reproducibility (%) [95% two-sided Wilson-score CI]	Repeatability (%) [95% two-sided Wilson-score CI]
1	Thyroid carcinoma	*CCDC6::RET*	1.37x	100.0 [86.2, 100.0]	100.0 [75.75, 100.0]
2	Thyroid carcinoma	*CCDC6::RET*	2.09x	100.0 [85.1, 100.0]	100.0^†^
3	Thyroid papillary carcinoma	*CCDC6::RET*	2.14x	100.0 [85.7, 100.0]	100.0 [74.1, 100.0]
4	Ovary serous carcinoma	*CCDC6::RET*	1.90x	100.0 [86.2, 100.0]	100.0 [75.78, 100.0]
5	Pancreas neuroendocrine carcinoma	*ERC1::RET*	1.50x	100.0 [85.7, 100.0]	100.0 [74.1, 100.0]
6	Lung adenocarcinoma	*KIF5B::RET*	1.76x	100.0 [85.7, 100.0]	100.0 [74.1, 100.0]
7	Lung adenocarcinoma	*KIF5B::RET*	2.18x	100.0 [86.2, 100.0]	100.0 [75.8, 100.0]
8	Lung adenocarcinoma	*KIF5B::RET*	1.48x	100.0 [86.2, 100.0]	100.0 [75.8, 100.0]
9	Colon adenocarcinoma	*NCOA4::RET*	2.41x	95.5 [78.2, 99.2]	90.0^†^
10	Colon adenocarcinoma	*NCOA4::RET*	1.53x	100.0 [86.2, 100.0]	100.0 [75.8, 100.0]
11	Colon adenocarcinoma	*NCOA4::RET*	2.49x	100.0 [86.2, 100.0]	100.0 [75.8, 100.0]
12	Colon adenocarcinoma	*NCOA4::RET*	2.45x	100.0 [86.2, 100.0]	100.0 [75.8, 100.0]
13	Colon adenocarcinoma	*PRPF19::RET*	1.41x	95.8 [79.8, 99.3]	91.7 [64.6, 98.5]
14	Colon adenocarcinoma	*TRIM24::RET*	2.10x	100.0 [86.2, 100.0]	100.0 [75.8, 100.0]

CI, confidence interval.

*The Fold LoD is the observed average chimeric reads across all valid replicates of the *RET* fusion within the sample divided by the LoD (8.75 chimeric reads).

^†^CIs were reported when the sample size was >10.

The precision of the F1CDx chimeric reads was assessed for the *RET* fusions by variance component analysis (VCA). Reproducibility of inter-run replicates (run on different plates under different conditions) and repeatability between intra-run replicates (run on the same plate under the same conditions) were assessed for three days nested within two different sites and two different reagent lots. The average chimeric reads, standard deviation (SD), and percent coefficient of variance (%CV) for reproducibility and repeatability are summarized for each *RET* fusion in **Table 4**.

**Table 4:** Reproducibility and repeatability for chimeric reads in *RET* fusions.

Sample	Cancer type	*RET* fusion	Observed average chimeric reads	Reproducibility		Repeatability	
				SD	%CV	SD	%CV
1	Thyroid carcinoma	*CCDC6::RET*	12.0	3.49	29.15	1.88	15.74
2	Thyroid carcinoma	*CCDC6::RET*	18.3	4.55	24.92	3.97	21.72
3	Thyroid papillary carcinoma	*CCDC6::RET*	18.7	5.17	27.61	4.86	25.95
4	Ovary serous carcinoma	*CCDC6::RET*	16.6	3.91	23.54	3.56	21.44
5	Pancreas neuroendocrine carcinoma	*ERC1::RET*	13.1	2.62	19.97	2.55	19.40
6	Lung adenocarcinoma	*KIF5B::RET*	15.4	5.69	36.87	4.08	26.43
7	Lung adenocarcinoma	*KIF5B::RET*	19.0	4.61	24.23	3.41	17.91
8	Lung adenocarcinoma	*KIF5B::RET*	13.0	4.34	33.48	4.34	33.48
9	Colon adenocarcinoma	*NCOA4::RET*	21.1	7.41	35.14	7.39	35.02
10	Colon adenocarcinoma	*NCOA4::RET*	13.4	5.13	38.37	3.71	27.77
11	Colon adenocarcinoma	*NCOA4::RET*	21.8	12.23	56.21	11.78	54.17
12	Colon adenocarcinoma	*NCOA4::RET*	21.4	5.98	27.94	5.98	27.94
13	Colon adenocarcinoma	*PRPF19::RET*	12.4	4.33	34.98	3.92	31.69
14	Colon adenocarcinoma	*TRIM24::RET*	18.4	4.43	24.11	2.99	16.29

The study demonstrated robust performance of the F1CDx assay for detecting *RET* fusions near the LoD in 14 solid tumor samples with at least 95% reproducibility, at least 90% repeatability, and the VCA assessment of chimeric reads for each *RET* fusion within the same samples.

### Orthogonal concordance to externally validated assay

An orthogonal concordance study was performed to demonstrate the concordance between the F1CDx assay and an evNGS assay for the detection of *RET* fusions in solid tumors. Among the total 410 samples that were included in the orthogonal concordance study, the following tumor types were included: 160 NSCLC samples, 100 thyroid cancer samples, and 150 other solid tumor samples. The other solid tumor samples included samples from colorectal (n=51), ovary (n=24), breast (n=20), pancreas (n=13), prostate (n=8), salivary gland (n=8), small intestine (n=3), endocrine-neuro (n=2), glioma (n=2), melanoma (n=2), skin (n=2), soft tissue sarcoma (n=2), biliary (n=1), cervix (n=1), GI-neuro (n=1), and unknown (n=10) cancer types. **Table 5** presents the *RET* fusion detection across the 410 samples between the F1CDx assay and the evNGS assay. Across 410 samples, 407 were valid by both assays and included in the analysis; one sample was not successful for the evNGS assay testing, and two additional samples were not successful for F1CDx testing (indicated as Unevaluable in **Table 5**). The PPA for *RET* fusion calls between F1CDx and evNGS was 100.0% (95% two-sided Wilson-score CI [97.4%, 100.0%]) and the NPA was 97.3% (95% two-sided Wilson-score CI [94.6%, 98.7%]). OPA was 98.3% (95% two-sided Wilson-score CI [96.5%, 99.2%]). The PPV for *RET* fusion calls between F1CDx and the evNGS assay was 95.4% (95% two-sided Wilson-score CI [90.9%, 97.8%]) and the NPV was 100.0% (95% two-sided Wilson-score CI [98.5%, 100.0%]).

**Table 5:** Contingency table for comparing *RET* fusion detection between F1CDx and evNGS assay.

Concordance betweenF1CDx and evNGS		evNGS			
	*RET* fusion status	*RET* Fusion+	*RET* Fusion (-)	Unevaluable	Total
F1CDx	*RET* Fusion (+)	146	7	1	154
	*RET* Fusion (-)	0	254	0	254
	Unevaluable	0	2	0	2
	Total	146	263	1	410

Seven samples had discordant *RET* fusion status between the F1CDx and evNGS test results. All discordant status samples were determined to be *RET* fusion-negative by the evNGS assay and *RET* fusion-positive by F1CDx. Of the seven discordant samples, four did not report *RET* fusions by evNGS due to low tumor content or low sample quality, one sample had a *RET* fusion detected in a biomarker-negative orientation by evNGS, and two samples did not have *RET* fusions detected by evNGS (*RET* fusion partner genes detected by F1CDx were *FXYD4* and *ZNF485*). For those seven *RET* fusions that were positive by F1CDx, all were in canonical orientation and the fusion partner genes were *FXYD4, NCOA4, NCOA4, ZNF485, PIBF1, KIF5B*, and *EML4*.

### Clinical bridging study

Among 243 pooled *RET* fusion-positive patients identified by CTA from the LIBRETTO-001 clinical trial evaluated for efficacy, 119 had sufficient residual material available for F1CDx testing. After testing, 87 patient samples had valid results from the F1CDx assay. Among 140 negative procured solid tumor samples selected by representative CTAs used in LIBRETTO-001 and then tested by F1CDx, 138 had valid results by the F1CDx assay. The analysis and corresponding results in this section considered any reported *RET* fusion that would be reported on a F1CDx report as a positive call; these fusions were not required to meet the more stringent criteria defined by the CDx device for biomarker positivity that were used in the CDx validation studies.

A contingency table with the concordance of *RET* fusion calls between the CTA tests and F1CDx test is provided in **Table 6**. The PPA and NPA (**Table 7**) were established as 90.80% (95% two-sided Wilson-score CI [82.89%, 95.27%]) and 100.00% (95% two-sided Wilson-score CI [97.29%, 100.00%]), respectively, after excluding unevaluable results (defined as samples that had invalid F1CDx test results or were not tested by F1CDx and indicated as Unevaluable in **Tables 6**, **8**). The overall percent agreement (OPA) was 96.44% (95% two-sided Wilson-score CI [93.14%, 98.19%]). The PPV and NPV (**Table 7**) were calculated using the PPA and NPA after adjusting for the *RET* prevalence (i.e., 0.6%, 7) of *RET* fusions among the intended use (IU) population due to enrichment bias resulting from *RET* fusion-positive and -negative sample selection by the CTAs. The bootstrap method was used to calculate the CI for the NPV, and because the PPV was 100%, bootstrapping was not applicable and the CI for the PPV was calculated with the Wilson-score method using prevalence-unadjusted counts.

**Table 6:** Contingency table for comparing *RET* fusion detection between F1CDx and the CTAs.

Concordance between F1CDx and CTA			CTA		
	*RET* fusion status	*RET* Fusion (+)	*RET* Fusion (-)	Total
F1CDx	*RET* Fusion (+)	79	0	79
	*RET* Fusion (-)*	8	138	146
	Unevaluable	156	2	158
	Total	243	140	383

*Included six samples that had a qualified F1CDx test report. A qualified report indicates that sensitivity for variant detection, including fusions, is reduced. In the clinical setting, this will be stated on the clinical report and that the test should be repeated.

**Table 7:** Concordance analysis results.

Agreement metric	Point estimate (%)	95% Two-sided CI (%)
PPA	90.80	[82.89, 95.27]
NPA	100.00	[97.29, 100.00]
Adjusted PPV	100.00	[95.36, 100.00]*
Adjusted NPV	99.94	[99.90, 99.98]^†^

*The Wilson-score method was used based on the prevalence-unadjusted PPV.

^†^CI was calculated by bootstrapping 2,000 times.

**Table 8:** Primary efficacy in the clinical bridging study subpopulations for *RET* fusion reporting.

Efficacy parameters	CTA+	F1CDx+|CTA+	F1CDx-|CTA+	F1CDx unevaluable or qualified F1CDx-|CTA+
Number of Subjects	243	79	2	162
Number of Responders (CR or PR)	139	55	0	84
ORR (%)	57.20	69.62	0.00	51.85
95% Two-Sided Wilson-Score CI (%)	[50.92, 63.26]	[58.77, 78.66]	N/A*	[44.20, 59.41]

*CIs were reported when the sample size was >10.

An investigation into the eight discordant samples (*RET* fusion positive by CTA and *RET* fusion negative by F1CDx) (**Table 9**) showed that most (6/8) had the *RET* fusion call, but it was removed due to potential sample contamination, triggering a qualified test report and therefore recommendation for reflex testing. Only two (2/8) samples did not show evidence of *RET* fusion detection by F1CDx. In both cases, the samples were different specimens and temporally different between the CTA test and F1CDx test, and neither subject was a responder to selpercatinib.

**Table 9:** Investigation into eight samples with discordant *RET* fusion calls between CTA and F1CDx.

Tumor type	CTA name	CTA fusion event	F1CDx report status	Discordance investigation	Known/likely pathogenic alterations reported by F1CDx
Colon	IBM Watson Genomics Core Test	*NCOA4::RET*	Pass	No *RET* fusion detected by pipeline. CTA and CDx tests tested different specimens.	• *APC* R213* • *TP53* R248Q • *APC* R876* • *SMAD4* loss
Colon	FoundationOne^®^Liquid CDx	*CCDC6::RET*	Pass	No *RET* fusion detected by pipeline. Different specimen type used for CTA test and specimen was temporally different from F1CDx test.	• *CTNNB1* R376C • *APC* K1363* • *TP53* C135R • *ERBB2* R678Q • *BCL2L1* amplification • *CDK4* amplification • *MYC* amplification
Pancreatic	Archer FusionPlex Custom Solid Panel	*NCOA4::RET*	Qualified^†^	*RET* fusion was removed due to contamination.	• *CDKN2A* P114H • *TP53* splice site 376-1G>A • *MYCN* amplification • *GATA6* amplification
NSCLC	FoundationOne CDx	*SLC12A2::RET*	Qualified^†^	*RET* fusion was removed due to contamination.	• *GNAS* R201H • *PIK3R1* R274fs*8 • *DNMT3A* splice site 2408 + 1G>T
NSCLC	Oncomine Focus Assay	*KIF5B::RET*	Qualified^†^	*RET* fusion was removed due to contamination.	• *SMARCA4* L976fs*43 • *CDH1* splice site 388-2A>T • *PMS2* E225fs*33 • *MRE11* E624fs*47 • *ATM* A2301fs*9 • *TNFAIP3* C158fs*58 • *MUTYH* splice site 892-2A>G • *ATM* splice site 8011-5_8011-1ATTAG>TTT • *EED* G69fs*18 • *ATRX* V1002fs*1 • *NBN* T226fs*5 • *CDK4* amplification • *MDM2* amplification
NSCLC	FoundationOne^®^	*KIF5B::RET*	Qualified^†^	*RET* fusion was removed due to contamination.	• *TP53* Q331* • *MAP2K4* Q316* • *PTEN* loss • *MTAP* loss • *CDKN2B* loss • *CDKN2A* loss
NSCLC	HapOnco 451 Gene Detection	*CCDC6::RET*	Qualified^†^	*RET* fusion was removed due to contamination.	• *TP53* L130V • *CDK4* amplification
NSCLC	FoundationOne	*KIF5B::RET*	Qualified^†^	*RET* fusion was removed due to contamination.	• *TP53* W146* • *NKX2–1* amplification

^†^ A qualified report indicates that sensitivity for variant detection, including fusions, is reduced. In the clinical setting, this will be stated on the clinical report and that the test should be repeated.

The ORR was calculated for the 243 pooled efficacy-evaluable CTA-positive patients and subsets by F1CDx *RET* fusion detection status (**Table 8**).

The ORR for the subset of *RET* fusion-positive subjects by F1CDx (F1CDx+|CTA+) was numerically higher than the CTA-positive (CTA+) population and other subgroups. Based on statistical methods in part by Li 2015, the ORR was estimated for the *RET* fusion-positive population as 69.62% (95% two-sided CI [59.48%, 79.76%], based on the variance of the estimated F1CDx-positive ORR) when identified by F1CDx. The point estimate of ORR estimated for the *RET* fusion-positive population identified by F1CDx is the same as the point estimate for the ORR in the F1CDx+|CTA+ population due to the prevalence-adjusted PPV being 100.00%.

## Discussion

Activating *RET* fusions have emerged as a biomarker for advanced cancer patients with solid tumors who may benefit from RTK inhibitors. In 2022, the FDA approved the tyrosine kinase inhibitor selpercatinib for the treatment of locally advanced or metastatic *RET*-fusion positive cancers, based on the results from the LIBRETTO-001 clinical trial. Here, we present the pan-tumor analytical and clinical validation of F1CDx, an NGS-based CGP assay, for identifying *RET* fusions in solid tumors. Analytical validation for *RET* fusions demonstrated that F1CDx showed a 0% false positive rate, >90% reproducibility and repeatability, a LoD of 8.75 chimeric reads as well as high concordance to an externally validated assay. Notably, the 90.80% PPA and 99.94% prevalence-adjusted NPV values are likely underestimated in the clinical bridging study because of sample quality issues; six of eight (6/8) discordances (positive by CTA but negative by F1CDx) were caused by removal of detected *RET* fusions in the F1CDx sample due to contamination, triggering a qualified test report. In addition, the clinical validity of *RET* fusion detection by F1CDx was further supported by patient outcomes from LIBRETTO-001, in which *RET* fusion-positive patients identified by F1CDx showed clinical benefit from selpercatinib treatment.

*RET* fusion status can be determined using multiple methods, including fluorescence *in situ* hybridization (FISH), polymerase-chain-reaction (PCR) assay, or NGS-based assays. In LIBRETTO-001, the CTA was initially performed locally using FISH, PCR, or NGS by certified molecular laboratories. In this retrospective clinical bridging study, F1CDx identified *RET*-fusion positive solid tumor patients who responded to selpercatinib, as evidenced by a numerically higher ORR in this subgroup. Notably, the ORR among patients whose *RET* fusion statuses were not available or F1CDx-|CTA+ with a qualified report from F1CDx was numerically lower than the overall cohort ORR.

Importantly, only two of eight (2/8) fully passing samples in this study failed to detect *RET* fusions by F1CDx despite being positive by CTA, and neither of these patients responded to selpercatinib. This discrepancy may reflect tumor and temporal heterogeneity, as the F1CDx and the CTA assays were conducted on different specimens and collected at different time points. These findings underscore the importance of specimen quality and timing in detection of actionable biomarkers when comparing two different tests.

Molecularly guided precision oncology and tissue-agnostic regulatory approvals have significantly reshaped the treatment landscape for solid tumors. Although *RET* fusions are associated with ~1-2% of lung and 5-10% of thyroid cancers, they are rare across other solid tumors (<1%) (7). A key advantage of a CGP approach like F1CDx is its ability to identify *RET* fusions, while preserving tissue and simultaneously testing for clinically actionable genomic alterations and complex biomarkers (13). The ability of comprehensive genomic profiling (CGP) to simultaneously assess multiple biomarkers is increasingly essential in precision oncology, where optimal treatment selection often depends on the integrated evaluation of diverse genomic alterations. In non-small cell lung cancer (NSCLC), for example, patients who test negative for RET fusions may harbor alternative actionable driver alterations, including mutations or rearrangements in EGFR, ALK, ROS1, NTRK, MET (exon 14 skipping), BRAF (V600E), or KRAS (G12C). Sequential single-gene testing strategies are inherently limited, as they may deplete available tumor tissue and prolong turnaround time, potentially delaying treatment initiation. In contrast, CGP enables parallel interrogation of multiple targets from a single specimen, preserving tissue and expediting results. Additionally, CGP provides assessment of genome-wide biomarkers that are not captured by single-gene assays. These include tumor mutational burden (TMB) and microsatellite instability (MSI), which may inform immunotherapy selection, as well as homologous recombination deficiency (HRD) signatures that can guide the use of PARP inhibitors and platinum-based therapies.

Another advantage of CGP (e.g. over FISH) is its ability to identify the fusion partner gene, which has important diagnostic implications. Knowledge of the fusion partner can improve diagnostic specificity, as certain partners are enriched in specific tumor types or histologic contexts and may support tumor classification, particularly in cases with ambiguous morphology or limited immunohistochemical data (e.g., *KIF5B* in non-small cell lung cancer and *CCDC6* or *NCOA4* in thyroid carcinoma). In addition, fusion partner identification increases confidence in the biological validity of the rearrangement by distinguishing oncogenic, in-frame events from potential artifacts or non-functional rearrangements, and NGS-based approaches further allow precise breakpoint characterization to refine functional annotation.

One limitation of this study is that the clinical patient population was restricted to patients enrolled in LIBRETTO-001-patients aged 18 years and older (or ≥12 years, where permitted) with limited information on F1CDx efficacy on pediatric patients < 12 years of age. Although pediatric patient age was not an exclusion criterion for the analytical validation and concordance studies, most participants were adults. A second limitation is that the LIBRETTO-001 population may not fully represent the broader population of patients with *RET* fusion-positive tumors identified by F1CDx. Accordingly, the clinical bridging study aimed to estimate the efficacy of selpercatinib, using efficacy data from LIBRETTO-001, among *RET* fusion-positive solid tumor patients when identified by F1CDx.

In conclusion, this study demonstrates the rigorous analytical and clinical performance of F1CDx in identifying *RET f*usions across solid tumors, supporting its tumor-agnostic FDA approval in 2023 and approval by Japan’s MHLW in 2024 as a CDx for selpercatinib. F1CDx is a robust, accurate, reliable, and FDA- and MHLW-approved, DNA-based CGP assay for the pan-tumor detection of *RET* fusions predictive of selpercatinib response. Importantly, the two CTA-positive but F1CDx-negative patients were non-responders, re-affirming the clinical utility. Similar CGP strategies may also be extended to liquid biopsies (14, 15), further enhancing accessibility and utility in clinical practice. Overall, our results fill a critical need in the validation of *RET* fusion testing strategies and support the use of F1CDx as a clinically reliable companion diagnostic. This study contributes to addressing an unmet medical need by enabling precise identification of *RET* fusion-positive cancer patients across diverse tumor types who may benefit from targeted therapy with selpercatinib.

## Data Availability

All relevant data were provided within the article. If needed, additional details may also be obtained by contacting the corresponding author.
